# A Perspective on Polo-Like Kinase-1 Inhibition for the Treatment of Rhabdomyosarcomas

**DOI:** 10.3389/fonc.2019.01271

**Published:** 2019-11-22

**Authors:** Susanne A. Gatz, Ewa Aladowicz, Michela Casanova, Julia C. Chisholm, Pamela R. Kearns, Simone Fulda, Birgit Geoerger, Beat W. Schäfer, Janet M. Shipley

**Affiliations:** ^1^Cancer Research UK Clinical Trials Unit (CRCTU), Institute of Cancer and Genomic Sciences, University of Birmingham, Birmingham, United Kingdom; ^2^Divisions of Molecular Pathology and Cancer Therapeutics, The Institute of Cancer Research, London, United Kingdom; ^3^Istituto Nazionale dei Tumori, Milan, Italy; ^4^Children and Young People's Unit, The Royal Marsden NHS Foundation Trust, London, United Kingdom; ^5^Institute for Experimental Cancer Research in Pediatrics, Goethe-University Frankfurt, Frankfurt, Germany; ^6^Gustave Roussy Cancer Campus, Department of Paediatric and Adolescent Oncology, Université Paris-Saclay, Villejuif, France; ^7^Department of Oncology and Children's Research Center, University Children's Hospital Zurich, Zurich, Switzerland

**Keywords:** polo-like kinase 1, PLK1 inhibitors, rhabdomyosarcomas, combination treatment, microtubule disruptors

## Abstract

Rhabdomyosarcomas are the most common pediatric soft tissue sarcoma and are a major cause of death from cancer in young patients requiring new treatment options to improve outcomes. High-risk patients include those with metastatic or relapsed disease and tumors with *PAX3-FOXO1* fusion genes that encode a potent transcription factor that drives tumourigenesis through transcriptional reprogramming. Polo-Like Kinase-1 (PLK1) is a serine/threonine kinase that phosphorylates a wide range of target substrates and alters their activity. PLK1 functions as a pleiotropic master regulator of mitosis and regulates DNA replication after stress. Taken together with high levels of expression that correlate with poor outcomes in many cancers, including rhabdomyosarcomas, it is an attractive therapeutic target. This is supported in rhabdomyosarcoma models by characterization of molecular and phenotypic effects of reducing and inhibiting PLK1, including changes to the PAX3-FOXO1 fusion protein. However, as tumor re-growth has been observed, combination strategies are required. Here we review preclinical evidence and consider biological rationale for PLK1 inhibition in combination with drugs that promote apoptosis, interfere with activity of PAX3-FOXO1 and are synergistic with microtubule-destabilizing drugs such as vincristine. The preclinical effects of low doses of the PLK1 inhibitor volasertib in combination with vincristine, which is widely used in rhabdomyosarcoma treatment, show particular promise in light of recent clinical data in the pediatric setting that support achievable volasertib doses predicted to be effective. Further development of novel therapeutic strategies including PLK1 inhibition may ultimately benefit young patients with rhabdomyosarcoma and other cancers.

## Introduction

Rhabdomyosarcomas (RMS) are the most common pediatric soft tissue sarcoma and are a major cause of death from cancer in young patients. There are two main histological subtypes of pediatric RMS; embryonal RMS accounting for ~70% of cases and alveolar RMS ~ 30% of RMS. Similar to other pediatric cancer, RMS has low levels of somatic genetic aberrations compared to most adult cancers (1). Embryonal RMS is associated with aneuploidy, particularly gain of chromosomes 8, 12, and 2, loss of heterozygosity or imprinting around the insulin growth factor 2 (IGF2) locus and mutations in genes encoding proteins involved in RAS pathway signaling. The majority of cases with alveolar histology are characterized by gene fusions between the *PAX3* or *PAX7* and *FOXO1* genes (2, 3). The *PAX3-FOXO1* fusion gene encodes a novel and potent transcription factor that drives tumourigenesis through transcriptional reprogramming, including upregulation of the transcription factor MYCN and receptor tyrosine kinases (4–6). Furthermore, the fusion protein in a complex with bromodomain containing protein 4 (BRD4) has been shown to establish super-enhancer regions associated with changes to histone modifications that markedly affect expression levels of particular genes (7).

Fusion gene positive RMS tends to be more aggressive and a higher proportion of cases present with metastatic disease than fusion negative RMS. Furthermore, the presence of the fusion gene has been identified in both retrospective and prospective analyses as a molecular marker of poor patient outcome that is superior to using histological classification for risk stratification (8–11). Based on these observations and similarities in gene expression profiling data (9, 12), fusion gene status has been incorporated into risk stratification in the current US protocol and will replace histology in the new protocol for RMS in Europe.

Current treatment for RMS is based on conventional chemotherapy, surgical resection, and radiotherapy. Despite treatment intensification, improvement in outcome has been disappointing with overall survival rates of 70% (www.ncin.org.uk/databriefings) and patients with metastatic or relapsed disease having dismal outcomes (13, 14). Treatments are associated with short and long-term side effects, which can be severe (15, 16). There is a clear unmet clinical need for novel, more effective and less toxic therapeutic strategies, especially for higher-risk RMS patients which includes all fusion gene positive cases. Potential therapeutic strategies centered on the role of the fusion protein are reviewed in detail elsewhere (17, 18). Here we focus on the identification, molecular understanding and effects of inhibiting Polo-Like Kinase-1 (PLK1) as a promising molecular target for therapy of RMS. PLK1 inhibitors both alone and in combination with other agents are considered, including the effects targeting PLK1 has on the PAX3-FOXO1 fusion protein.

## PLK1 Function

PLK1 is the most extensively studied of five members of the polo-like family of serine/threonine kinases and has a wide range of target substrates that it phosphorylates. It is primarily known for operating as a pleiotropic master regulator of the cell cycle from entry into mitosis to the initiation of cytokinesis. This includes regulating the activity of proteins involved in establishing centromeres, initiating spindle checkpoint signaling and coordinating the activity of the spindle checkpoint, as reviewed in detail elsewhere (19, 20). High levels of PLK1 expression are generally restricted to rapidly dividing cells such as those during embryogenesis and in hair follicles. Significantly, many types of cancer, including pediatric tumors, also express high PLK1 levels. Overexpression is correlated with poor prognosis in several tumor types and reduction of PLK1 expression or its inhibition results in a failure of cell cycle regulatory mechanisms that can lead to subsequent apoptosis of cancer cell lines and xenograft models, including those of pediatric solid tumors (21–24).

In addition to the peak activation of PLK1 in the G2/M phase of the cell cycle, expression and basal activity starts early in S phase with PLK1 regulating DNA replication, notably under stress. Phosphorylation of ORC2 by PLK1 is reported to promote DNA replication (25) and is associated with resistance to gemcitabine (an inhibitor of DNA replication) in pancreatic tumor cells (26). PLK1 activity is also reported to be involved with resistance to doxorubicin (21). The CDK-PLK1 axis targets RAD9, a DNA checkpoint sensor protein, that minimizes checkpoint response (27). Therefore, PLK1 functions by complex mechanisms to regulate DNA replication after stress as well as mitosis, in ways that may be relevant to responses to cancer treatment and tumor development, including in RMS.

## PLK1 in RMS

The levels of expression of PLK1 in pediatric cancers and RMS are comparable to high levels seen in many adult cancers and are even higher in pediatric cancer cell lines, including those representing RMS (28). The highest levels in primary alveolar RMS assessed by immunohistochemistry correlated with poor event and overall survival (*n* = 49). High PLK1 levels also correlated with the expression of a downstream target of PAX3-FOXO1, AP2beta, but not MIB1, a marker of proliferation (29).

A genome wide RNA interference screen reducing expression levels of kinases in RMS, Ewing sarcoma, and neuroblastoma cell lines identified PLK1 as one of the most important kinases for cell proliferation and survival (22). In cancer cell lines and xenograft models including fusion positive RMS it has been noted that reduction of PLK1 expression or its inhibition leads to mitotic arrest which can lead to apoptosis (22–24).

It has been known for some time that the PAX3-FOXO1 fusion protein can be phosphorylated by kinases and that phosphorylation contributes to DNA binding, transcriptional and oncogenic activity (30, 31). To identify druggable upstream regulatory kinases of the PAX3-FOXO1 fusion protein a system to read out activity of the fusion protein was combined with a double screening strategy (29). A luciferase-based reporter system was constructed using the AP2beta promotor fragment, as AP2beta is a transcriptional target of PAX3-FOXO1, and introduced into a fusion positive cell line (RH4). These cells were subjected to siRNA and small molecule libraries representative of the kinome to identify upstream regulators of the fusion protein. Reducing PLK1 transcription and treatment with the PLK1 inhibitor BI2536 were key hits that were validated in two further cell lines and shown to impact on PAX3-FOXO1 activity through assessment of additional known downstream target genes of PAX3-FOXO1 (PIPOX, FGFR4, and CDH3). In addition, expression of AP2beta significantly correlated with expression of PLK1 in primary RMS, consistent with PLK1 levels affecting levels and/or activity of PAX3-FOXO1 in patients (29). Co-immunoprecipitation experiments established direct interaction between PAX3-FOXO1 and PLK1. Further experiments, including use of expression mutant c wild-type PAX3-FOXO1 in a fusion-negative cell line, demonstrated that serine 503 in the PAX3-FOXO1 fusion protein, corresponding to serine 322 in the FOXO1 protein, is a PLK1 specific phosphorylation site. As this phosphorylation site in FOXO1 is also present in PAX7-FOXO1 it is expected that PLK1 inhibition will also affect this fusion protein. Furthermore, PLK1 inhibition has been shown to result in decreased expression of the MYCN oncogene (29). This may be via PAX3-FOXO1 transcriptionally regulating MYCN expression (4, 29) and/or PLK1 regulating the stability of MYCN, which itself is upregulated by MYCN (32). Reduction of MYCN in RMS has been shown to result in reduced cell proliferation and apoptosis (6) and therefore effects of PLK1 inhibition could uniquely include those from targeting MYCN and the PAX-FOXO1 fusion protein in addition to impacting on the cell cycle.

In addition, PLK1 is known to activate other proteins in AML and ALL including PI3K and mTOR (33). The PI3K/AKT/mTOR pathway has been shown to be highly active and to play a critical role in maintaining fusion positive and negative RMS cell proliferation (34), although a link with PLK1 activity has not yet been demonstrated in RMS.

## Preclinical Data for PLK1 Inhibitors in RMS

Volasertib (BI6727), is a derivative of BI2536, and is a highly potent ATP competitive PLK1 inhibitor. Two different studies have investigated the impact of volasertib on pediatric cancer cell lines in 2D culture (28, 35). Data indicate that overall, pediatric cancer cell lines show some but variable sensitivities to PLK1 inhibition. Between 4 and 6 cell lines representing acute lymphocytic leukaemias (ALL), medulloblastomas, osteosarcomas, Ewing sarcomas, neuroblastomas and RMS showed GI50s in the range of 4–40 nM in the majority of cell lines tested (28, 35). The lowest GI50s in both studies were in PAX3-FOXO1 positive RMS cell lines which had values (Supplementary Table 1) less than those seen in acute myeloid leukemia (AML) cell line models (*in vitro* GI50s ranging from 9 to 36nM) (36, 37). These and other data for AML supported clinical trials of volasertib in leukemia [as reviewed in (38)].

The degradation of the fusion protein and reduction in transcription of targets of the fusion protein have been noted by treatment of fusion positive RMS cells with 15 nM BI2536 or 20 nM volasertib (29) and could contribute to the growth inhibitory effects of PLK1 inhibition and antitumorigenic effects of PAX3-FOXO1. However, Abbou et al. (28) also assessed LC50 (Lethal Concentration to kill 50% of cells) and demonstrated that whilst all ALL had LC50s <80 nM, most osteosarcoma had LC50s >2,500 nM and the other tumor specific cell lines, including RMS had examples of both low and high LC50s (Supplementary Table 1).

*In vivo*, one of four fusion gene positive RMS cell line xenografts (RH30R) showed complete remission after 30 mg/kg volasertib treatments (35). Volasertib also resulted in complete and partial tumor regressions of established xenograft tumors of the fusion positive RMS-01 cell line in another study (28). *In vivo* treatment of the cell line xenografts RH4 and RH13 with the PLK1 inhibitor BI2536 also led to regression of established xenograft tumors in mice that were shown to express less of the fusion-protein target proteins AP2beta and P-Cadherin vs. untreated tumors, consistent with suppression of fusion protein activity (29). However, the effects observed in mice are at higher doses of volasertib than tolerated in humans (35, 36). To achieve cell death at lower doses and counter the prospect of resistant cells arising, including the potential indicated for RMS to evolve independence from the fusion proteins (39, 40), PLK1 inhibition alone seems unlikely to be curative for RMS patients. Therefore, strategies for combining a PLK1 inhibitor with chemotherapy or a novel agent are required and preclinical work on potential combinations have already been published (as summarized in Table 1).

**Table 1:** Summary of rhabdomyosarcoma (RMS) preclinical studies for PLK1 inhibitors combined with other drugs.

**PLK1 inhibitor**	**Combination partner**	**RMS cell line models**	**Result (CI = Combination Index)**	**References**
BI2536	Vincristine	*In vitro* RD, TE381.T (fusion-negative) Primary RMS cells (fusion-negative)	CI <1.0 and synergy in various assays	(41)
		Rh30 (fusion-positive)	CI <1.0	(41)
Volasertib	Vincristine	RD, TE381.T	CI <1.0	(41)
Volasertib	Vincristine	RMS1 (fusion-positive)	CI <1.0	(28)
BI2536	Vincristine	*in vivo* RD cells grown in chorioallantoic membrane model	At least additive effects	(41)
Volasertib	Vincristine	RD xenograft s.c. in mice	At least additive effects	(41)
BI2536	Vinblastine/vinorelbine	RD (fusion-negative)	CI <1.0	(41)
BI2536	Eribulin	RD, TE381.T (fusion-negative), patient-derived fusion negative cells	CI <1.0, synergy in several assays	(42)
		Rh30 (fusion-positive)	CI > 1.0	(42)
BI2536	Paclitaxel	RD (fusion-negative)	CI > 1.0	(41)
BI2536	Doxorubicin	RD, TE381.T (fusion-negative), Rh30 (fusion-positive)	CI > 1.0	(41)
Volasertib	Etoposide	RMS1 (fusion-positive)	CI > 1.0	(28)

## Combination Strategies With PLK1 Inhibition for RMS Treatment

As the data suggest that single agent PLK1 inhibition may be inadequate to treat RMS, it is important to consider combinations based on biological rationale. The potential contribution of targeting the fusion protein via PLK1 inhibition is consistent with the known effects of modulating PAX3-FOXO1 expression levels on growth arrest, myogenic differentiation, migration and invasion as well as potentially apoptosis (17). NOXA expression in PAX3-FOXO1 expressing cells has been associated with susceptibility to apoptosis through BH3 mimetic treatment (43). Similarly, we have recently identified NOXA as a mediator of apoptosis that is induced by downregulation of PAX3-FOXO1creating sensitivity to the inhibitor navitoclax that targets BCL-XL and other members of the BCL-2 family of proteins (44). High levels of the anti-apoptotic protein BCL-XL have been noted in glioma stem cells resistant to volasertib (45). This and related apoptotic mechanisms may contribute to the variable responses in the growth and death of RMS cells to volasertib (Supplementary Table 1) and be amenable to therapeutic exploitation with drugs such as navitoclax.

The roles of PLK1 in regulating key aspects of mitosis means that inhibition leads to defective chromosome segregation that can cause DNA damage and a DNA damage response (46). Consistent with this, volasertib is known to induce DNA damage and consequent activation of the ATM-CHK1/CHK2 checkpoint pathway and G2/M arrest, for example in small cell lung cancer (47). Enhancing volasertib induced G2/M arrest with additional DNA damage through radiation led to apoptosis in glioma stem cells. However, the combination of volasertib and etoposide (a topoisomerase II inhibitor) or BI2536 and doxorubicin (a topoisomerase II inhibitor as well as alkylating agent) in RMS cells were antagonistic potentially due to etoposide/ doxorubicin induced G2 arrest prior to realization of the effects of volasertib on mitosis (28, 41, 48) (Table 1). Treating with etoposide after volasertib reduced antagonism and highlights the need to consider how best to schedule drugs (28).

The recruitment of BRD4 into a complex with the PAX3-FOXO1 protein in RMS is essential for high transcriptional activity at super-enhancers in collaboration with other transcription factors known to be critical in RMS (such as MYCN, MYOD1, and MYOG) and creates a dependency on BRD4 activity associated with vulnerability to BET inhibitors in fusion positive RMS (7). In addition, the BET inhibitor JQ1 is reported to impact on angiogenesis in pediatric sarcomas including RMS (49). However, low sub-micromolar concentrations of the BET inhibitor JQ1 had little effect on cell death of fusion positive and negative RMS cells although combination strategies may be beneficial (50). Interestingly, synergistic activity has been reported in AML with the BET inhibitor BI894999 and volasertib, both *in vitro* and *in vivo* (37). The effectiveness of this combination has also been recently indicated for RMS (51).

PLK1 inhibition (volasertib and BI2536) has been shown in several studies to be synergistic with the microtubule-destabilizing drugs vincristine, vinblastine, vinorelbine, and eribulin) in RMS (28, 41, 42) although synergy was not found using paclitaxel, possibly due to its different mode of action (41, 52). *In vitro* data with combination indices below 0.9 document synergy between volasertib and vincristine in fusion gene positive cell lines (28, 41). Fusion negative RMS cell lines and a patient derived cell line model, also showed synergy when treated with BI2536 or volasertib in combination with vincristine *in vitro* and at least additive effects *in vivo* (28, 41) (Table 1). The volasertib dose used by Hugle *et al.*, was very low compared to the other published xenograft data (5 mg/kg once per week for 5 weeks vs. 30 mg or 40 mg//kg/week for 3 or 5 weeks (28, 41). This effectiveness at a low dose holds promise to be achievable in patients based on pharmacokinetic data (53).

**Figure 1:**
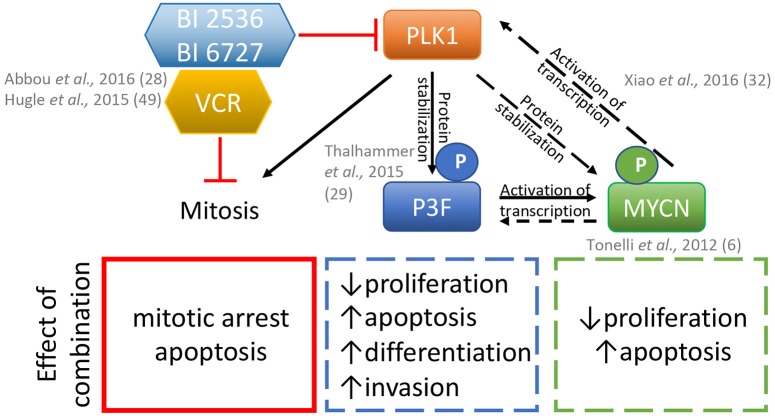
Model for the effects of the combination of PLK1 inhibitors BI2536 or BI6727 (volasertib) plus the microtubule disrupter vincristine (VCR) on rhabdomyosarcoma cells. Inhibition of PLK1 and vincristine disrupts mitosis which in combination can synergistically lead to apoptosis. PLK1 inhibition also affects the phosphorylation of the PAX3-FOXO1 (P3F) protein and MYCN either indirectly via P3F loss and/or directly via its phosphorylation status, and may additionally contribute to the effects of these inhibitors in fusion positive rhabdomyosarcomas. Dashed and solid lines, represent proven and plausible links and effects in rhabdomyosarcoma models, respectively.

Mechanistically, the combination of BI2536 and vincristine triggered mitotic arrest with subsequent mitochondrial apoptosis induced via inactivation of anti-apoptotic BCL2 proteins and caspase dependent and independent pathways. The key molecule of the caspase dependent pathway was identified as Myeloid Cell Leukemia−1 (MCL-1) (41). These data indicate an important fusion gene independent, cooperative effect of the volasertib-vincristine combination treatment on tumor growth. Vincristine is a highly active drug currently used in standard first line combination chemotherapy for RMS and is also frequently reused in the relapse setting (54, 55). Clinical testing by adding a PLK1 inhibitor to vincristine treatment of patients therefore represents an attractive strategy, as summarized in Figure 1.

## Clinical Translation and Concluding Comments

The preclinical data reviewed here, with the notable exception of inclusion of a patient derived xenograft in one study (41), use long-established cell lines which have been shown to express higher levels of PLK1 than primary tumors (28). This highlights the need to test model systems that better recapitulate primary tumors in preclinical testing, including use of 3D *in vitro* modeling and patient derived xenografts. Whilst the mechanistic effects of targeting PLK1 on the fusion protein are attractive, the preclinical RMS data reviewed here for volasertib and BI2536 as single agents with tumor re-growth and innate as well as potentially emerging resistance to cell death, indicates that combination approaches are necessary. Mechanistic considerations of the effects of agent combinations and the doses and their timings are critical, particularly with targets like PLK1 that are involved in mitotic regulation.

Volasertib has been tested in the clinic for years as single agent in different combinations in adult patients with acute myeloid leukemia (the aimed licensing indication for volasertib development) as well as in various solid tumors, and although exceptional responses have been seen, overall results were disappointing (38). Moreover, the phase III POLO-AML-2 trial (NCT01721876) in elderly patients which investigated low dose cytarabine plus placebo vs. low dose cytarabine plus volasertib, did not show an objective response rate that was not statistically significant in the volasertib arm and there were toxicity issues that led to a trend toward inferior overall survival (38). Whilst the studies in adult leukemia and solid tumors focused on the specific role of PLK1 inhibition on mitotic function (36), here describe new mechanisms of actions for this class of inhibitors that likely contribute to the specific sensitivity of RMS models. Importantly, volasertib has recently successfully completed Phase I as single agent in children with leukemia and refractory solid tumors (NCT01971476) (53) and a recommended Phase II dose (RP2D) was identified that lies above the RP2D for adult patients. In this clinical study, comparable with adult studies, the main toxicity was myelotoxicity manifesting as thrombocytopenia, neutropenia and febrile neutropenia and, also consistent with the data in adult patients, the pharmacokinetic data suggest plasma levels are in the dose range used to investigate synergy with vincristine in preclinical RMS models (28, 41).

The preclinical effects of vincristine in combination with volasertib observed in fusion negative RMS models at low volasertib/ BI2536 doses appear strong. Whilst the molecular mechanism affecting the fusion protein are striking with single agent BI2536/volasertib, testing fusion gene positive RMS models with the volasertib/vincristine combination has been limited and requires further assessment (Table 1). However, taken together with widespread use of vincristine in the treatment of newly diagnosed and relapsed RMS and the likely non-overlapping toxicities of vincristine and volasertib, this combination looks feasible and holds particular immediate promise to clinically assess in both fusion gene positive and negative RMS. If limited proof-of-concept clinical testing demonstrates tolerability of doses and activity, more intensive backbone chemotherapy and/or other targeted agents may subsequently be investigated that ultimately lead to improving outcomes for RMS patients.

## Author Contributions

SG, EA, and JS drafted the manuscript. All authors contributed to revisions, proof-reading, and approved the submitted version.

### Conflict of Interest

The authors declare that the research was conducted in the absence of any commercial or financial relationships that could be construed as a potential conflict of interest.
