# Supplementary material for: A Perspective on Polo-Like Kinase-1 Inhibition for the Treatment of Rhabdomyosarcomas
Source: Front Oncol. 2019 Nov 22;9:1271. doi: 10.3389/fonc.2019.01271 (PMC6882953; doi:10.3389/fonc.2019.01271)
Supplement: Supplementary file 1 [file Table_1.docx]

**SUPPLEMENTARY TABLE 1.** **GI50 (Growth Inhibitory) and LC50 (Lethal Concentration) doses for fusion positive fp-RMS and fusion negative fn-RMS cell lines reported in two separate studies (bold indicates doses that may be considered sensitive – see text).**

| Drug | Cell line | Tumour  type | GI50 mean  nmol/l Abbou et al 2016 (ref28) | LC50 mean  nmol/l Abbou et al 2016 (28) | GI50 median  nmol/l Gorlick et al 2014 (35) |
| --- | --- | --- | --- | --- | --- |
| BI6727  Volasertib | RH30 | **fp-RMS** | **21.7+/-17.8** | **31+/-20.6** | **8.2** |
|  | RH41 | **fp-RMS** | **3.7+/-0.41** | 3,876+/-111 | **6.9** |
|  | RMS-01 | **fp-RMS** | **3.56+/-0.36** | **9.40+/-0.29** |  |
|  | RD | **fn-RMS** | **26+/-1.49** | **50.2+/-11.1** | **16.5** |
|  | RH18 | fn-RMS | 219+/-229 | 6,158+/-1,174 | 135.2 |
|  | RMS-YM | fn-RMS | 444+/-11.4 | 3,370+/-697 |  |
